# Automatically Generated Smartphone Data in Young Patients With Newly Diagnosed Bipolar Disorder and Healthy Controls

**DOI:** 10.3389/fpsyt.2021.559954

**Published:** 2021-08-25

**Authors:** Sigurd Melbye, Sharleny Stanislaus, Maj Vinberg, Mads Frost, Jakob Eyvind Bardram, Lars Vedel Kessing, Maria Faurholt-Jepsen

**Affiliations:** ^1^The Copenhagen Affective Disorder Research Center, Rigshospitalet, Copenhagen, Denmark; ^2^Faculty of Health and Medical Sciences, University of Copenhagen, Copenhagen, Denmark; ^3^Psychiatric Research Unit, Psychiatric Center North Zealand, Hillerød, Denmark; ^4^Monsenso ApS, Copenhagen, Denmark; ^5^Department of Health Technology, Technical University of Denmark, Kongens Lyngby, Denmark

**Keywords:** bipolar disorder, smartphones, sensor data, child and adolescent psychiatry, activity, social activity

## Abstract

**Background:** Smartphones may facilitate continuous and fine-grained monitoring of behavioral activities *via* automatically generated data and could prove to be especially valuable in monitoring illness activity in young patients with bipolar disorder (BD), who often present with rapid changes in mood and related symptoms. The present pilot study in young patients with newly diagnosed BD and healthy controls (HC) aimed to (1) validate automatically generated smartphone data reflecting physical and social activity and phone usage against validated clinical rating scales and questionnaires; (2) investigate differences in automatically generated smartphone data between young patients with newly diagnosed BD and HC; and (3) investigate associations between automatically generated smartphone data and smartphone-based self-monitored mood and activity in young patients with newly diagnosed BD.

**Methods:** A total of 40 young patients with newly diagnosed BD and 21 HC aged 15–25 years provided daily automatically generated smartphone data for 3–779 days [median (IQR) = 140 (11.5–268.5)], in addition to daily smartphone-based self-monitoring of activity and mood. All participants were assessed with clinical rating scales.

**Results:** (1) The number of outgoing phone calls was positively associated with scores on the Young Mania Rating Scale and subitems concerning activity and speech. The number of missed calls (*p* = 0.015) and the number of outgoing text messages (*p* = 0.017) were positively associated with the level of psychomotor agitation according to the Hamilton Depression Rating scale subitem 9. (2) Young patients with newly diagnosed BD had a higher number of incoming calls compared with HC (BD: mean = 1.419, 95% CI: 1.162, 1.677; HC: mean = 0.972, 95% CI: 0.637, 1.308; *p* = 0.043) and lower self-monitored mood and activity (*p*'s < 0.001). (3) Smartphone-based self-monitored mood and activity were positively associated with step counts and the number of outgoing calls, respectively (*p*'s < 0.001).

**Conclusion:** Automatically generated data on physical and social activity and phone usage seem to reflect symptoms. These data differ between young patients with newly diagnosed BD and HC and reflect changes in illness activity in young patients with BD. Automatically generated smartphone-based data could be a useful clinical tool in diagnosing and monitoring illness activity in young patients with BD.

## Introduction

Bipolar disorder (BD) is a serious, recurrent, and disabling disorder often with an onset of symptoms during a young age (1). In addition to fluctuations in mood, BD is characterized by fluctuations in behavioral, social, and physical activity with alterations both during affective episodes and between episodes (2). As of today, there are no blood tests, radiologic findings, or other biomarkers to assist clinical decision making in symptom monitoring and diagnostic evaluations; hence, early interventions rely on clinical evaluations often made with large intervals between outpatient visits, making monitoring of symptoms vulnerable to potential recall bias (3).

Diagnostic work in children and adolescents with psychiatric disorders is especially challenging as it is often characterized by unspecific prodromal symptoms (4). Correct diagnosis and interventions are crucial in the early stages of BD (5). The clinical presentation of children and adolescents with BD is characterized by a more continuous course of affective dysregulation, with episodes of depression and (hypo)mania lasting for hours rather than days or weeks, as in adult-onset BD (6). Also, (hypo)mania is characterized by irritability more than elation (7).

Smartphones, equipped with sensors, such as accelerometers, are widely used all over the world, with 45% of people in the world owning a smartphone (8). This allows for smartphones to make potentially meaningful clinical data out of behavioral activity. Prior research has shown automatically generated smartphone data to give an accurate reflection of behavioral activity associated with fluctuations in BD in adults (9–13).

For many young people, smartphone interaction is a significant part of their everyday life and important for social interaction (14). Thus, smartphones would be widely available to many young people as a diagnostic and monitoring tool.

However, for a diagnostic tool to be useful, it needs foremost to be able to differentiate between patients and healthy controls (HC). Recent studies have shown that smartphone-based self-monitored data represent symptom burden according to clinical ratings and are also able to differentiate between adult patients with newly diagnosed BD and HC (9, 10, 12, 15). Nonetheless, self-monitoring demands that users be devoted to daily self-monitoring of their mood, activity, etc. Dedication to perform the monitoring is vulnerable to attrition, and adherence often decreases over time (16). This issue is avoidable using automatically generated smartphone data, which do not depend on daily self-monitoring. If automatically generated smartphone data are associated with self-monitored data, it can somewhat compensate for the decrease of adherence in self-monitored data, in addition to supplementing it with more fine-grained information.

Prior research has found automatically generated smartphone data to be useful, feasible, and valid for adult patients with BD (9, 10, 12, 15, 17–20). In a recent systematic review conducted by the authors investigating the use of smartphones in self-monitoring and treatment of adolescents and young adult patients with psychiatric disorders (21), we identified two studies collecting automatically generated smartphone data only (in patients with depression and early psychosis), but none of these studies included automatically generated smartphone data in their analyses (22, 23). To conclude, to date, no studies on automatically generated smartphone data on young patients with BD and HC have been published.

## Objectives

The present pilot study aimed to (1) validate automatically generated smartphone data reflecting physical and social activity and phone usage against validated clinical ratings and questionnaires in young patients with newly diagnosed BD and HC; (2) investigate differences in automatically generated smartphone data reflecting physical and social activity and phone usage between young patients with newly diagnosed BD and HC; and (3) investigate associations between automatically generated smartphone data reflecting physical and social activity and phone usage and smartphone-based self-monitored activity and mood in young patients with newly diagnosed BD.

Based on prior research on adults with BD, we hypothesized that (1) automatically generated smartphone data reflecting physical and social activity and phone usage would be associated with validated clinical ratings and questionnaires, among young patients with newly diagnosed BD and HC; (2) automatically generated smartphone data reflecting physical and social activity and phone usage differ between young patients with newly diagnosed BD and HC with lower scores in physical and social activity in BD than in the HC group; (3) automatically generated smartphone data reflecting physical and social activity and phone usage would be associated with smartphone-based self-monitored activity and mood, with positive associations between smartphone-based self-monitored activity and mood, and automatically generated smartphone data on physical and social activity, respectively, in young patients with newly diagnosed BD.

## Materials and Methods

The participants included in the present study were recruited as part of the Bipolar Illness Onset study (the BIO study) (24), a longitudinal observational study including patients with newly diagnosed BD, their unaffected relatives, and HC (25, 26). In the BIO study, all participants underwent a clinical assessment, combined with blood tests, MRI scan, and cognitive tests at baseline in addition to annual visits.

### Study Design, Settings, and Participants

Among the participants in the BIO study, we recruited individuals newly diagnosed with BD and control persons without a personal or psychiatric family history, aged 25 years or younger at the time of inclusion (HC). Participants newly diagnosed with BD were recruited from the Copenhagen Affective Disorder Clinic at Rigshospitalet in Copenhagen, Denmark. The Copenhagen Affective Disorders Clinic is a specialized clinic that offers a 2-year course of treatment to everyone newly diagnosed with BD in the larger region of Copenhagen over the age of 18. We also included young patients with newly diagnosed BD under the age of 18, from the Child and Adolescent Mental Health Center in Copenhagen. We recruited HC among blood donors from the Blood Bank at Rigshospitalet. The exclusion criterion for the latter was a history of a psychiatric disorder requiring treatment, personally or in a first-degree relative.

### Diagnostic Assessment

All participants underwent a diagnostic interview using Schedules for Clinical Assessment in Neuropsychiatry (SCAN) (27), during the baseline interview, to ensure that participants fulfilled the inclusion criteria for the respective groups. Trained researchers performed the SCAN interviews. For young patients with newly diagnosed BD, baseline interviews were performed by PhD students in medicine or psychology; for HC, some of the SCAN interviews were performed by medical or psychology students.

### Baseline Interview and Follow-Up

The baseline interview consisted of a collection of general information about educational and work status in addition to diagnostic and clinical assessments. Information about time for onset of symptoms, diagnosis, start of treatment, and number and duration of affective episodes was collected among the young patients with newly diagnosed BD. All participants also completed a questionnaire addressing physical activity. After baseline, participants attended an annual follow-up interview as well as interviews every time they had a change from one affective episode to another, from an affective episode to euthymic stage, or from a euthymic stage to an affective episode.

### Clinical Ratings

At baseline and follow-up interviews, the following rating scales were used: the severity of depressive symptoms was assessed using the 17-item Hamilton Depression Rating Scale (HAMD) (28, 29); the severity of manic symptoms was assessed using the Young Mania Rating Scale (YMRS) (30); and the level of functioning was assessed using the Functioning Assessment Short Test (FAST) (31), which is a test developed explicitly for BD and addresses six areas of functioning (autonomy, occupational functioning, cognitive functioning, financial issues, interpersonal relationship, and leisure time). All 24 items are rated from 0 (no difficulties) to 3 (severe difficulties) and assess the last 2 weeks up to the rating. FAST has been validated against the Global Assessment of Functioning scale (GAF) and has a high test–retest reliability (31).

### Questionnaire

The participants completed the International Physical Activity Questionnaire Short Form (IPAQ) at each visit with the researchers. In the IPAQ, participants report how many minutes of physical activity in different intensities (vigorous, moderate, and walking) they had the prior week; these are then converted to metabolic equivalent task (MET) minutes per week, which then add up to a total MET score (32). MET is equivalent to kilocalories for a 60-kg person; we adjusted for weight for each participant to express the last week's activity in kcal/week (33).

### Smartphone-Based Monitoring

The Monsenso system is a smartphone-based monitoring system with a collection of automatically generated smartphone data as mobility, activity, and phone usage using the telephones sensors, in addition to daily self-monitoring of, e.g., mood, sleep, and activity (34, 35). The system is available for both Android and iOS; however, the collection of automatically generated smartphone data is only available on smartphones using the Android operating system; thus, in this article, only participants using Android phones were included. Every participant was registered in the Monsenso system before the time of inclusion and instructed to start using the system 3 days before the baseline interview. The participants installed the app in their private phone, or we offered to loan an Android smartphone (LG Nexus 5) to participants not owning a phone. In this study, only one participant utilized a borrowed phone. We asked the young patients with newly diagnosed BD to use the system for at least 3 months, and the HC were asked to use it for at least 1 month. Participants were reminded to start using the system again when they were booked for follow-up interviews. As HAMD and YMRS capture symptoms from 3 days prior to the interview, we chose to include only participants who provided at least 3 days of smartphone data.

The automatically generated smartphone data collected daily by the Monsenso system were step counts; the total number of steps during a 24-h period detected by the accelerometer in the phone, reflecting the physical activity; the number of incoming and outgoing text messages during a 24-h period, reflecting social activity; duration and number of incoming and outgoing calls and the number of missed calls during a 24-h period, reflecting social activity; and the number of seconds the screen was on (screen time) and the number of times the screen was turned on, reflecting the smartphone usage.

For self-monitoring, all participants scored their daily level of activity and their mood, reflecting how good or how bad their day had been on a 7-point scale (−3, −2, −1, 0, 1, 2, 3). Additionally, young patients with newly diagnosed BD scored their affective state on a 9-point scale with scores from depressive to manic, which is a more fine-grained scale (−3, −2, −1, −0.5, 0, 0.5, 1, 2, 3) (34).

### Statistical Analyses

Hypotheses and statistical analyses for the present study were defined *a priori*. For continuous variables, we used linear mixed effect models to investigate between-group differences in the mean regarding background characteristics. We used chi-square tests for between-group differences of categorical data. For analyses concerning aims 1–3, for each measure of interest, we employed a linear mixed effect model, which accommodates both the variation of the variables of interest within young patients with newly diagnosed BD (intra-individual variation) and between individuals (inter-individual variation). For participants with data available from both baseline and up to nine follow-up visits, a linear mixed effect model analysis to account for repeated measurements within each participant was employed. All participants were identified with a unique ID number. For aim 1, we used a linear mixed effect model to analyze the association between automatically generated smartphone data, as the dependent variable, and scores from clinical rating scales and questionnaires. For these analyses, averages of automatically generated smartphone data for the current day and 3 days before ratings with the HAMD and the YMRS, 7 days prior for the IPAQ, and 14 days prior for the FAST were calculated. For aim 2, we used a linear mixed effect model to investigate the between-group difference in daily automatically generated smartphone data between young patients with newly diagnosed BD and HC, from every observation available. For aim 3, we used a linear mixed effect model to analyze the associations between smartphone-based self-monitored mood and activity, as the dependent variable, and automatically generated smartphone data on physical and social activity and phone usage, respectively. Individual ID number was added as a random factor for all analyses. For aim 1, we included pooled data available from all participants. For aim 3, we included data available from young patients with newly diagnosed BD only. Analyses were conducted first in an unadjusted model and secondly in models adjusted for age and sex, by adding sex and age (as a numeric covariate) as fixed factors.

As no prior studies have investigated differences in automatically generated smartphone data between young patients with newly diagnosed BD and HC, we were not able to perform statistical power analyses prior to the study. Since data were collected as part of a larger longitudinal observational study, the sample size was defined according to this. As this is the first study on automatically generated smartphone data in young patients with BD and, in this way, the study is explorative by nature, we did not correct for multiple analyses. We checked model assumptions by visually utilizing residuals and QQ plots for each of the statistical analyses. SPSS version 25 (Statistical Package for the Social Sciences) was used for all analyses. A *p*-value ≤ 0.05 was considered statistically significant.

## Results

**Figure 1:**
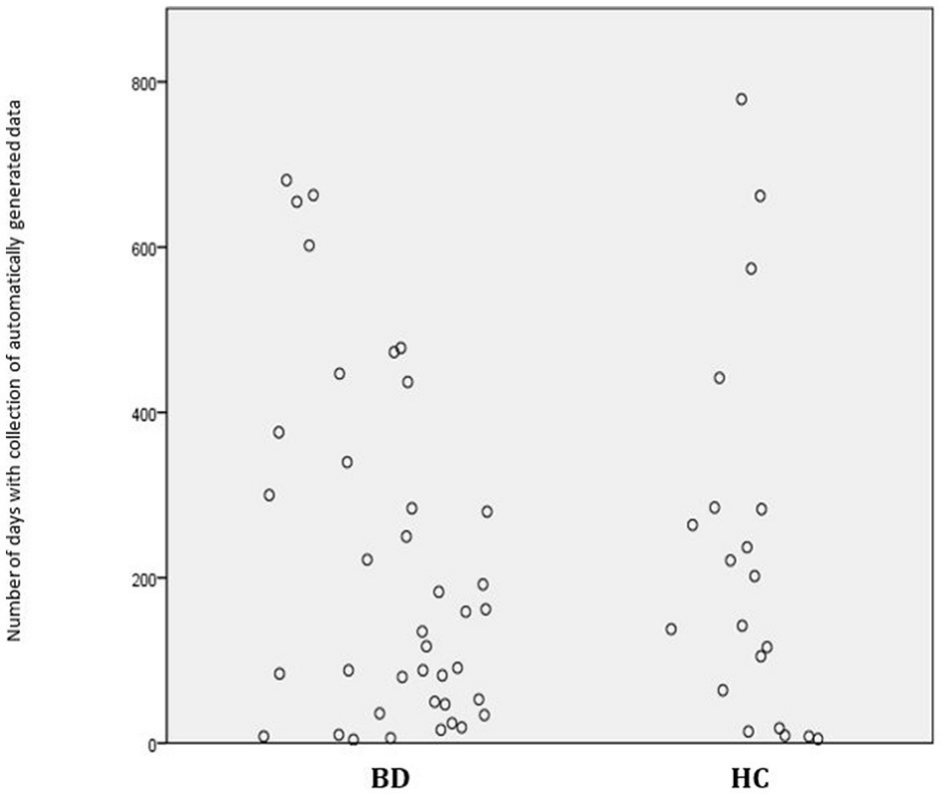
The number of days with collection of automatically generated smartphone data in young patients with newly diagnosed bipolar disorder (BD) and healthy control individuals (HC). Each circle represents a participant.

**Figure 2:**
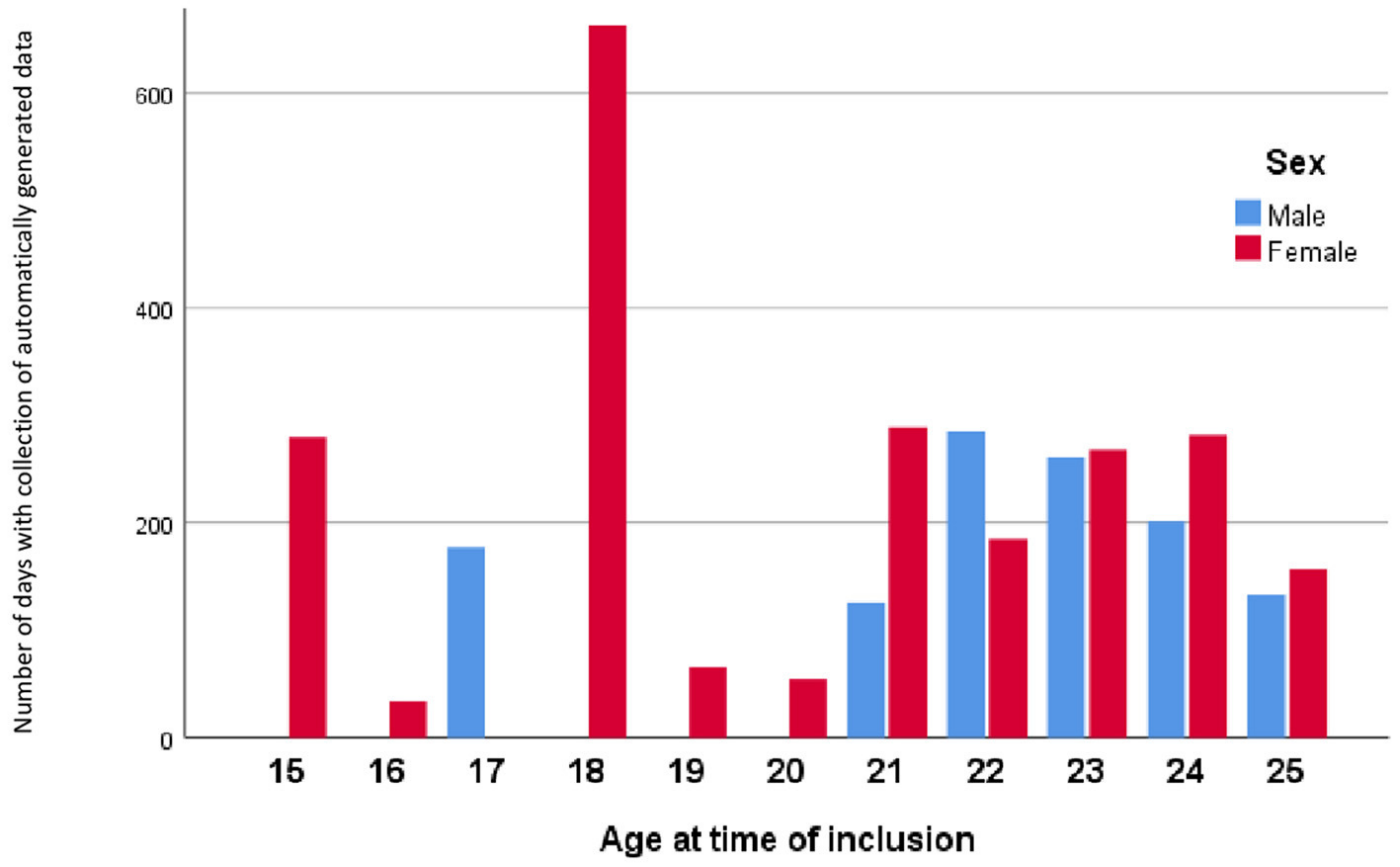
The mean number of days with collection of automatically generated smart phone data grouped by age and sex, for all participants pooled (*n* = 61).

The present pilot study included a total of 40 young patients with newly diagnosed BD and 21 HC aged 25 years or younger by the time of inclusion and who provided automatically generated smartphone data. The HC group was statistically significantly older than the BD group (BD: mean = 21.6, 95% CI: 20.9, 22.3; HC: mean = 23.1, 95% CI: 22.2, 24.0; *p* = 0.012), the groups did not differ on other background characteristics (Table 1). Furthermore, all models were adjusted for age, and age was an insignificant covariate in all the analyses comparing BD with HC. The number of days where participants performed smartphone-based self-monitoring varied from 3 to 779 days [median (IQR) = 140 (11.5–268.5)] and added up to a total of 12,827 days. A total of 80% of the young patients with newly diagnosed BD and 71% of the HC had automatically generated data available for more than 1 month (Figures 1, 2). The number of visits for each participant varied from one to nine visits. A total of 27 participants had more than one visit. There were a total number of 77 visits with clinical assessment and automatically generated smartphone data from the 3 days prior to the visit (52 in the BD group and 25 in the HC group). Among the 52 in the BD group, 28 were during remission, 14 during a depressive episode, 6 during a hypomania, and 2 during a manic or mixed episode.

**Table 1:** Demographics and background characteristics of young patients with newly diagnosed BD and HC, at baseline, *N* = 61.

	**BD**	**HC**	**BD vs. HC** ***p***
Participants	40	21	
Age, years	21.6 (20.9, 22.3)	23.1 (22.2, 24.0)	**0.012**
Female, % (*n*)	72.5 (29)	61.9 (13)	0.40
Education, years	10.4 (5.8, 15.0)	15.0 (8.6, 21.3)	0.25
Full-time employment, % (*n*)	5.0 (2)	9.5 (2)	0.51
Student, % (*n*)	65.0 (26)	76.2 (16)	0.37
HAMD-17^a^	10.9 (8.7, 13.1)	1.2 (−1.8, 4.2)	**<0.001**
YMRS^b^	3.88 (2.65, 5.10)	0.48 (−1.21, 2.16)	**0.002**
FAST, total score^c^	19.83 (16.12, 23.53)	1.48 (−3.64, 6.60)	**<0.001**
IPAQ total, kcal/week^c^	2,489.10	2,437.92	0.90
Bipolar disorder II, % (*n*)	72.5 (29)	–	–
Age of onset, years	15 (12.87–17.13)	–	–
Illness duration, years^e^	5.5 (2.0–9.0)	–	–
Years of untreated BD^f^	1 (0.0–2.0)	–	–
No. of depressive episodes	4 (0.00–8.75)	–	–
No. of hypomanic episodes	3 (0.50–5.50)	–	–
No. of manic episodes	0 (0.00–0.75)	–	–
No. of mixed episodes	0 (0.00–1.00)	–	–
No. of total episodes	9.50 (4.13–14.88)	–	–

*Continuous variables are presented as mean (SD) or median [interquartile range], and p-values are calculated based on differences in the mean between the two groups using linear mixed effect models. Categorical data are presented as % (n), and p-values are calculated using chi-square tests*.

a *HAMD-17, 17-item Hamilton Depression Rating Scale*.

b *YMRS, Young Mania Rating Scale*.

c *FAST, Functional Assessment Short Test*.

d *IPAQ, International Physical Activity Questionnaire adjusted for weight and converted to kcal/week*.

e *llIness duration was defined as the time from the first episode to the time of inclusion*.

f *Years of untreated BD was defined as the time from the first mania, hypomania, or mixed episode to time of diagnosis*.

*Bold values indicate statistical significance (p-value ≤ 0.05)*.

### Validity of Automatically Generated Smartphone Data Against Clinical Ratings and Questionnaires

These results can be seen in Table 2.

**Table 2:** Associations between automatically generated smartphone data reflecting physical activity, social activity, and phone usage and clinical ratings and questionnaires in young patients with newly diagnosed BD and HC, from 77 clinical interviews (52 with BD and 25 with HC)^a^.

	**Number of observations**	***B***	**95% CI**	***p***
**Step count (number/day)^b^**				
HAMD 17-item total score^c^	43	−4.26	(−154.90, 146.39)	0.96
HAMD subitem 8**^d^**	43	−201.36	(−1,791.15, 1,388.44)	0.81
HAMD subitem 9**^e^**	43	817.71	(−1,313.08, 2,948.50)	0.44
YMRS total^f^	43	21.92	(−301.81, 345.65)	0.89
YMRS subitem 2**^g^**	43	371.92	(−1,768.61, 2,512.45)	0.73
YMRS subitem 6**^h^**	43	−38.41	(−1,482.68, 1,405.87)	0.96
FAST**^i^**	49	−26.76	(−82.50, 28.97)	0.34
IPAQ**^j^**	37	0.037	(−0.42, 0.49)	0.87
**Screen time (min/day)^b^**				
HAMD 17-item total score^c^	76	−0.44	(−4.72, 3.84)	0.84
HAMD subitem 8**^d^**	76	0.77	(−41.07, 42.61)	0.97
HAMD subitem 9**^e^**	76	−10.05	(−71.13, 51.02)	0.74
YMRS total^f^	76	−2.82	(−9.98, 4.35)	0.43
YMRS subitem 2**^g^**	76	−39.08	(−94.08, 15.91)	0.16
YMRS subitem 6**^h^**	76	−14.98	(−49.62, 19.67)	0.39
FAST**^i^**	77	0.99	(−0.51, 2.50)	0.19
IPAQ**^j^**	60	−0.009	(−0.022, 0.003)	0.12
**Screen on (number/day)^b^**				
HAMD 17-item total score^c^	77	−0.57	(−1.98, 0.84)	0.42
HAMD subitem 8**^d^**	77	−11.33	(−24.62, 1.97)	0.093
HAMD subitem 9**^e^**	77	−9.18	(−29.41, 11.05)	0.37
YMRS total^f^	77	0.61	(−1.97, 3.19)	0.64
YMRS subitem 2**^g^**	77	6.14	(−12.73, 24.99)	0.52
YMRS subitem 6**^h^**	77	0.39	(−11.28, 12.05)	0.95
FAST**^i^**	77	−0.12	(−0.73, 0.50)	0.70
IPAQ**^j^**	61	0.001	(−0.003, 0.005)	0.47
**Incoming calls (number/day)^b^**				
HAMD 17-item total score^c^	63	0.040	(0.005, 0.074)	**0.024**
HAMD subitem 8**^d^**	63	0.36	(0.025, 0.70)	**0.036**
HAMD subitem 9**^e^**	63	0.79	(0.30, 1.27)	**0.002**
YMRS total^f^	63	0.062	(−0.012, 0.14)	0.095
YMRS subitem 2**^g^**	63	0.38	(−0.15, 0.91)	0.16
YMRS subitem 6**^h^**	63	0.23	(−0.064, 0.53)	0.12
FAST**^i^**	65	0.014	(−0.002, 0.029)	0.077
IPAQ**^j^**	52	−3.307e−5	(−0.0001, 9.977e−5)	0.62
**Outgoing calls (number/day)^b^**				
HAMD 17-item total score^c^	63	0.031	(−0.029, 0.091)	0.30
HAMD subitem 8**^d^**	63	0.25	(−0.33, 0.83)	0.39
HAMD subitem 9**^e^**	63	1.024	(0.21, 1.84)	**0.015**
YMRS total^f^	63	0.18	(0.092, 0.26)	**<0.001**
YMRS subitem 2**^g^**	63	1.11	(0.43, 1.78)	**0.003**
YMRS subitem 6**^h^**	63	0.79	(0.48, 1.10)	**<0.001**
FAST**^i^**	65	0.007	(−0.026, 0.039)	0.69
IPAQ**^j^**	52	0.0001	(−5.488e−5, 0.0003)	0.14
**Missed calls (number/day)^b^**				
HAMD 17-item total score^c^	63	0.014	(−0.009, 0.038)	0.22
HAMD subitem 8**^d^**	63	0.11	(−0.12, 0.34)	0.33
HAMD subitem 9**^e^**	63	0.41	(0.083, 0.73)	**0.015**
YMRS total^f^	63	0.031	(−0.019; 0.080)	0.21
YMRS sub-item 2**^g^**	63	−0.012	(−0.36; 0.34)	0.95
YMRS sub-item 6**^h^**	63	0.16	(−0.038; 0.367)	0.11
FAST**^i^**	65	0.003	(−0.008; 0.014)	0.58
IPAQ**^j^**	52	8.844e-6	(−8.658e-5; 0.0001)	0.85
**Duration of phone calls (minutes/day)^b^**				
HAMD 17-item total score^c^	63	0.22	(−0.20; 0.63)	0.30
HAMD sub-item 8**^d^**	63	2.71	(−1.18; 6.59)	0.17
HAMD sub-item 9**^e^**	63	2.56	(−3.48; 8.61)	0.40
YMRS total^f^	63	0.009	(−0.78; 0.80)	0.98
YMRS sub-item 2**^g^**	63	−0.15	(−5.72; 5.42)	0.96
YMRS sub-item 6**^h^**	63	−0.58	(−3.76; 2.60)	0.71
FAST**^i^**	65	0.12	(−0.087; 0.32)	0.25
IPAQ**^j^**	52	0.0008	(−0.003; 0.001)	0.39
**Incoming text messages (number/day)^b^**				
HAMD 17-item total score^c^	62	0.13	(−0.036; 0.29)	0.12
HAMD sub-item 8**^d^**	62	1.49	(−0.15; 3.12)	0.075
HAMD sub-item 9**^e^**	62	3.26	(0.88; 5.64)	**0.008**
YMRS total^f^	62	0.20	(−0.57; 0.17)	0.29
YMRS sub-item 2**^g^**	62	−1.53	(−4.41; 1.34)	0.29
YMRS sub-item 6**^h^**	62	−0.73	(−2.30; 0.84)	0.35
FAST**^i^**	64	0.031	(−0.017; 0.078)	0.20
IPAQ**^j^**	50	8.056e-5	(−0.0004; 0.0006)	0.75
**Outgoing text messages (number/day)^b^**				
HAMD 17-item total score^c^	62	0.10	(−0.061; 0.26)	0.22
HAMD sub-item 8**^d^**	62	1.21	(−0.40; 2.82)	0.14
HAMD sub-item 9**^e^**	62	2.88	(0.53; 5.23)	**0.017**
YMRS total^f^	62	−0.19	(−0.56; 0.17)	0.29
YMRS sub-item 2**^g^**	62	−1.61	(−4.41; 1.19)	0.25
YMRS sub-item 6**^h^**	62	−0.75	(−2.29; 0.78)	0.33
FAST**^i^**	64	0.021	(−0.032; 0.075)	0.42
IPAQ**^j^**	50	5.224e-5	(−0.0005; 0.0006)	0.85

a *Models adjusted for age and gender*.

b *Averages of automatically generated smartphone data were calculated for the current day and 3 days before ratings with the HAMD and the YMRS, 14 days prior for the FAST rating, and 7 days prior for the IPAQ*.

c *Hamilton Depression Rating Scale (HAMD) 17-item version total score*.

d *HAMD sub-item 8—level of psychomotor retardation for the past 3 days*.

e *HAMD sub-item 9—level of psychomotor agitation for the past 3 days*.

f *Young Mania Rating Scale (YMRS) total score*.

g *YMRS sub-item 2—level of increased motor activity for the past 2 days*.

h *YMRS sub-item 6—increased talkativeness for the past 2 days*.

i *FAST, The Functional Assessment Short Test total score*.

j *IPAQ, The Physical Activity Questionnaire—short form total score calculated to be expressed in kcal/week*.

*Bold values indicate statistical significance (p-value ≤ 0.05)*.

As can be seen in Table 2, the number of incoming calls was statistically significantly positively associated with the HAMD total score (*B* = 0.040; 95% CI: 0.005, 0.074; *p* = 0.024), the HAMD subitem 8 (*B* = 0.36; 95% CI: 0.025, 0.70; *p* = 0.036), and the HAMD subitem 9 (*B* = 0.79; 95% CI: 0.30, 1.27; *p* = 0.002). Thus, for every increase of 1 point in the HAMD total score, there was a 0.04 increase in the number of incoming calls.

As can be seen in Table 2, the number of outgoing calls was statistically significantly positively associated with YMRS total score (*B* = 0.18; 95% CI: 0.092, 0.26; *p* < 0.001), the YMRS subitem 2 (*B* = 1.11; 95% CI: 0.43, 1.78; *p* = 0.003), the YMRS subitem 6 (*B* = 0.79; 95% CI: 0.48, 1.10; *p* < 0.001), and the HAMD subitem 9 (*B* = 1.024, 95% CI: 0.21, 1.84; *p* = 0.015). Thus, for every increase of 1 point in the YMRS total score, there was a 0.18 increase in the number of incoming calls.

The number of missed calls was statistically significantly positively associated with the HAMD subitem 9 (*B* = 0.41; 95% CI: 0.083, 0.73; *p* = 0.015). Thus, for every increase of 1 point in the HAMD subitem 9 score, there was a 0.41-point increase in the number of missing calls. The number of incoming text messages was statistically significantly positively associated with the HAMD subitem 9 (*B* = 3.26; 95% CI: 0.88, 5.64; *p* = 0.008), and the same was the case between the number of outgoing text messages and the HAMD subitem 9 (*B* = 2.88; 95% CI: 0.53, 5.23; *p* = 0.017). Thus, for every increase of 1 point in the HAMD subitem 9 score, there was a 3.26-point increase in the number of incoming text messages and a 2.88-point increase in the number of outgoing text messages. For the associations between the number of incoming text messages and scores on the clinical rating scales, age was also a significant covariate. We found no associations between screen time, the number of step counts, the number of times the screen was turned on, total screen time, and scores on clinical ratings or questionnaires.

### Differences in Smartphone Data Between Young Patients With Newly Diagnosed BD and HC

These results can be seen in Table 3.

**Table 3:** Differences in automatically generated smartphone data reflecting mobility, social activities and phone usage between young patients with newly diagnosed BD (*n* = 40), and healthy controls (*n* = 21), (*N* = 61).

	**BD**			**HC**			**BD/HC**
	**Number of observations**	**Mean**	**95% CI**	**Number of observations**	**Mean**	**95% CI**	***P***
**Automatically generated smartphone data**							
Step count (steps/day)	6,649	4,561.99	(3,206.22; 5,917.77)	2,977	5,459.78	(3,628.93; 7,290.64)	0.44
Screen time (minutes/day)	8,137	220.14	(175.10; 265.19)	4,547	157.63	(99.89; 215.38)	0.096
Screen on (number/day)	8,256	77.94	(58.40; 97.48)	4,568	64.45	(39.39; 89.51)	0.40
Call duration (minutes/day)	6,521	16.12	(11.58; 20.67)	3,188	8.70	(2.77; 14.63)	0.056
Incoming calls (number/day)	6,521	1.42	(1.16; 1.68)	3,188	0.97	(0.64; 1.31)	**0.043**
Outgoing calls (number/day)	6,521	2.32	(1.87; 2.77)	3,188	1.81	(1.23; 2.40)	0.18
Missed calls (number/day)	6,521	0.90	(0.73; 1.06)	3,188	0.72	(0.51; 0.93)	0.19
Incoming text-messages (number/day)	6,447	4.65	(3.44; 5.87)	3,729	4.60	(3.02; 6.20)	0.96
Outgoing text-messages (number/day)	6,447	3.52	(1.99; 5.03)	3,729	3.68	(1.70; 5.66)	0.90
**Smartphone-based self-monitored data**							
Activity score	4,742	−0.007	(−0.22; 0.21)	2,764	0.64	(0.38; 0.91)	**<0.001**
Mood	4,796	0.21	(−0.042; 0.46)	2,779	1.60	(1.28; 1.92)	**<0.001**
**Clinical ratings**							
HAMD 17-item total score**^a^**	76	8.51	(6.94; 10.09)	39	0.84	(−1.23; 2.91)	**<0.001**
HAMD sub-item 8**^b^**	76	0.37	(0.20; 0.53)	39	0.003	(−0.21; 0.22)	**0.009**
HAMD sub-item 9**^c^**	76	0.29	(0.17; 0.41)	39	0.034	(−0.12; 0.19)	**0.010**
YMRS total score^d^	76	4.11	(2.99; 5.24)	39	0.38	(−1.09; 1.85)	**<0.001**
YMRS sub-item 2**^e^**	76	0.39	(0.17; 0.61)	39	0.042	(−0.24; 0.33)	0.057
YMRS sub-item 6**^f^**	76	0.72	(0.47; 0.96)	39	−0.026	(−0.35; 0.30)	**<0.001**
FAST total score**^g^**	76	17.37	(14.39; 20.35)	39	0.33	(−3.57; 4.24)	**<0.001**
**Questionnaire**							
IPAQ total score**^h^**	72	3476.58	(2645.07; 4308.10)	36	2787.90	(1761.84; 3813.97)	0.31

*In total 12,827 days with collection of automatically generated smartphone data (BD: 8,258 days, HC: 4,569 days)**^i^***.

a *Hamilton Depression Rating Scale (HAMD) 17-item total score*.

b *HAMD Sub-item 8—level of psychomotor retardation*.

c *HAMD Sub-item 9—level of psychomotor agitation*.

d *Young Mania Rating Scale total score*.

e *YMRS Sub-item 2—level of increased motor activity*.

f *YMRS Sub-item 6—increased talkativeness*.

g *FAST, The Functional Assessment Short Test total score*.

h *IPAQ, The Physical Activity Questionnaire—short form total score calculated to be expressed in kcal/week*.

i *Adjusted for age and sex*.

*Bold values indicate statistical significance (p-value ≤ 0.05)*.

As can be seen, there was a statistically significant difference in the number of incoming calls between young patients with newly diagnosed BD and HC (BD: mean = 1.42, 95% CI: 1.16, 1.68; HC: mean = 0.97, 95% CI: 0.64, 1.31; *p* = 0.043). There were no other statistically significant differences in automatically generated smartphone data between young patients with newly diagnosed BD and HC. Furthermore, there was a statistically significant difference in smartphone-based self-monitored mood as well as activity between young patients with newly diagnosed BD and HC (mood: BD mean = 0.21, 95% CI: −0.42, 0.46; HC mean = 1.60, 95% CI: 1.28, 1.92; *p* < 0.001).

As can be seen, young patients with newly diagnosed BD had a statistically significant higher score on the HAMD, YMRS, and FAST compared with HC (results can be found in Table 3).

### Associations Between Automatically Generated Smartphone Data and Smartphone-Based Self-Monitoring

These results can be seen in Table 4.

**Table 4:** Associations between automatically generated smartphone data reflecting physical and social activity, and smartphone-based self-monitored activity and mood in young patients with newly diagnosed BD, (*N* = 40), from a total of 3,804 days with collection of both automatically generated smartphone data and smartphone-based self-monitored data^a^.

	**Number of observations**	***B***	**95% CI**	***p***
**Step count (number/day)**				
Smartphone-based self-monitored activity^b^	2,762	347.41	(233.06; 461.77)	**<0.001**
Smartphone-based self-monitored mood^c^	2,786	367.48	(119.61; 615.35)	**0.004**
**Screen time (min/day)**				
Smartphone-based self-monitored activity^b^	3,678	−12.05	(−16.70; −7.41)	**<0.001**
Smartphone-based self-monitored mood^c^	3,705	−15.87	(−24.79; −6.94)	**<0.001**
**Screen on (number/day)**				
Smartphone-based self-monitored activity^b^	3,756	7.62	(6.37; 8.87)	**<0.001**
Smartphone-based self-monitored mood^c^	3,782	10.49	(8.07; 12.90)	**<0.001**
**Call duration (min/day)**				
Smartphone-based self-monitored activity^b^	2,469	−1.52	(−2.42; −0.62)	**0.001**
Smartphone-based self-monitored mood^c^	2,482	−0.14	(−1.86; 1.57)	0.87
**Incoming calls (number/day)**				
Smartphone-based self-monitored activity^b^	2,469	−0.022	(−0.070; 0.027)	0.38
Smartphone-based self-monitored mood^c^	2,482	−0.017	(−0.11; 0.075)	0.72
**Outgoing calls (number/day)**				
Smartphone-based self-monitored activity^b^	2,469	0.21	(0.12; 0.29)	**<0.001**
Smartphone-based self-monitored mood^c^	2,482	−0.032	(−0.19; 0.13)	0.70
**Missed calls (number/day)**				
Smartphone-based self-monitored activity^b^	2,469	−0.021	(−0.064; 0.022)	0.33
Smartphone-based self-monitored mood^c^	2,482	−0.11	(−0.19; −0.032)	**0.006**
**Incoming text-messages (number/day)**				
Smartphone-based self-monitored activity^b^	2,468	0.24	(0.033; 0.44)	**0.023**
Smartphone-based self-monitored mood^c^	2,486	−0.089	(−0.46; 0.28)	0.64
**Outgoing text-messages (number/day)**				
Smartphone-based self-monitored activity^b^	2,469	0.20	(−0.018; 0.41)	0.072
Smartphone-based self-monitored mood^c^	2,482	0.017	(−0.38; 0.42)	0.93

a *Adjusted for age and gender*.

b *Smartphone-based self-monitored activity rated on a scale from −3 to +3*.

c *Smartphone-based self-monitored mood rated on a 9-point scale from −3 to +3*.

*Bold values indicate statistical significance (p-value ≤ 0.05)*.

As can be seen in Table 4, there was a statistically significant positive association between smartphone-based self-monitored activity and automatically generated data on step count (*B* = 347.41; 95% CI: 233.06, 461.77; *p* < 0.001), the number of time the screen was turned on (*B* = 7.62; 95% CI: 6.37, 8.87; *p* < 0.001), number of outgoing calls (*B* = 0.21; 95% CI: 0.12, 0.29; *p* < 0.001), and the number of incoming text messages (*B* = 0.24; 95% CI: 0.033, 0.44; *p* = 0.023). Furthermore, there was a statistically significantly negative association between smartphone-based self-monitored activity and automatically generated data on screen time (*B* = −12.05; 95% CI: −16.70, −7.41; *p* < 0.001) and call duration (*B* = −1.52; 95% CI: −2.42, −0.62; *p* = 0.001). Thus, for every increase of 347 increase in step count, there was 1-point increase in smartphone self-monitored activity.

There was a statistically significant positive association between smartphone-based self-monitored mood and automatically generated data on step count (*B* = 367.48; 95% CI: 119.61, 615.35; *p* = 0.004) and numbers of time the screen was turned on (*B* = 10.49; 95% CI: 8.07, 12.90; *p* < 0.001). There was a statistically significant negative association between smartphone-based self-monitored mood and automatically generated data on screen time (*B* = −15.87; 95% CI: −24.79, −6.94, *p* < 0.001). Thus, for every increase of 367 in step count, there is a 1-point increase in smartphone-based self-monitored mood.

## Discussion

For the first time, the present pilot study investigated the use of automatically generated smartphone data collected in young patients with newly diagnosed BD and HC. Intriguingly, as hypothesized, automatically generated smartphone data on social activity was associated with clinically evaluated depressive and manic symptoms. Furthermore, as hypothesized, smartphone-based self-monitored mood, activity, automatically generated smartphone data on social activity (number of incoming calls/day), and clinically evaluated symptoms differed between young patients with newly diagnosed BD and HC. However, in contrast with our hypotheses, there were no other differences in automatically generated smartphone data between young patients with newly diagnosed BD and HC. In addition, as hypothesized, automatically generated smartphone data on physical activity and phone usage were associated with smartphone-based self-monitored mood and activity in young patients with newly diagnosed BD.

### Validity of Automatically Generated Smartphone Data Against Clinical Ratings and Questionnaires

The findings that automatically generated smartphone data on social activity were associated with clinically evaluated depressive and manic symptoms are in line with prior studies on adult patients with BD (9–13).

However, in the present study, we only found this association between social activity, reflected by the number of calls and text messages, and total scores and subitem scores on the HAMD and the YMRS. A possible reason for this could be that the participants included in the present study did not present with severe symptoms during the clinical evaluations, and thus, the associations may differ during more severe states. Also, a possible explanation could be that young patients with newly diagnosed BD may present with more complex clinical presentation with unspecific prodromal symptoms (4) or a more continuous course of affective dysregulation, with episodes of depression and (hypo)mania lasting for hours rather than days or weeks, as in adult-onset BD (7). Further, in contrast to prior studies including adult patients with BD, the participants included in the present study were rather young, and it may be that this particular population may use smartphones for communication in different ways than older populations do, and thus, changes may not be captured with the automatically generated smartphone data included in the present study (36). We found no statistically significant associations between scores on FAST and automatically generated smartphone data. This may be a type II error due to few high scores on FAST.

### Differences in Smartphone Data Between Young Patients With Newly Diagnosed BD and HC

The findings that smartphone-based self-monitored mood, activity, and automatically generated smartphone data on social activity (number of incoming calls/day) differed between young patients with newly diagnosed BD and HC are in line with prior studies on adult patients with BD (10) and may reflect activation of the social network in young patients with newly diagnosed BD. Potentially automatically generated smartphone data on social activity could facilitate identification of BD in young people. Future studies including unaffected first-degree relatives could provide interesting knowledge on early changes in communicative activities and potentially be a useful supplementary diagnostic tool and facilitate early intervention, as it potentially allows for identification of prodromal symptoms which sometimes patients with BD during early stages of illness have difficulties identifying themselves (37).

### Associations Between Automatically Generated Smartphone Data and Smartphone-Based Self-Monitoring

The findings that automatically generated smartphone data on physical activity and phone usage was associated with smartphone-based self-monitored mood and activity in young patients with newly diagnosed BD are also in line with prior studies on adult patients with BD (9–13, 20). In the present study, we found no association between automatically generated smartphone data on social activity and smartphone-based self-monitored mood. This could be due to the fact that young people tend to use alternative smartphone-based applications for social communication over the traditional call and text messages that were collected in the present study (36). However, we included information on the amount of time on the smartphone (screen time), which would include time spent on other messaging applications.

In the present study, we chose a naturalistic approach where the participants used their own smartphones as they would naturally; hence, there are missing data from when participants, i.e., turned off their phone, left it at home, or deactivated the app. Due to the nature of automatically generated smartphone-based data, we cannot say anything about the time periods where data were not available (missing data). However, since most people carry their smartphone with them during most of the day and use it in most of their online communications, we find that the results from the present study are valid. Overall, results from the present study confirm that automatically generated smartphone data may be used to monitor illness activity in young patients with BD unobtrusively during naturalistic settings between outpatient visits in a fine-grained and valid manner.

### Limitations

Firstly, due to a large portion of iPhone users among the participants included in the BIO study, the number of participants using Android phones and thus being eligible for the present study was relatively limited. Therefore, the results from the present study should be interpreted with caution, as negative findings may be due to type II errors. It is possible that inclusion of a larger sample size and inclusion of a larger control group would have revealed other associations and differences. However, the present study is the first of its kind and therefore hypotheses generating.

Secondly, few young patients with newly diagnosed BD were clinically evaluated during severe depressive and manic episodes; this will lower the probability of finding associations between automatically generated smartphone data and clinically evaluated symptoms reflected by clinical rating scales. Future studies, with a higher number of established affective episodes, could do classification modeling investigating differences in smartphone data during euthymic and affective episodes. Thirdly, as the participants used their own smartphone, there was no standardized platform for data collection, possibly leading to heterogeneity in the data collected. Fourthly, several of the young patients with newly diagnosed BD were using psychotropic medicine; this was not accounted for in the analyses. Fifthly, the HC in the present study were recruited among blood donors, possibly representing a “super healthy” population (38, 39). Nevertheless, the blood donors included in this study were recruited from the same catchment area as patients with BD; they did not differ in educational or work status form patients, and they were not granted economic compensation for participating. Alternative methods for recruiting control groups include using advertisements and the Danish Civil Registration System. However, both methods have relatively low participation response rates and a high risk of selection bias. Taken together, we find that our control group represents the most reasonable and assessable control group for this study. Lastly, as the present study was not a randomized controlled trial, we were not able to investigate potential adverse effects or harms of the smartphone-based monitoring. Future randomized controlled trials could investigate this important aspect further.

### Advantages

All young patients with newly diagnosed BD in the present study were diagnosed at a specialized mood disorder clinic, and the diagnosis or lack of diagnoses was verified for all participants with a SCAN interview conducted by trained PhD students in medicine or psychology. Additionally, all participants were assessed using clinically validated observer-based rating scales with the HAMD, the YMRS, and the FAST. The smartphone-based system used in the present study is well-validated, is useful, and fulfills the safety of data storage and privacy requirements. Although the collection of automatically generated data works on Android led to a smaller population in this study, it is an advantage regarding global generalizability, as Android is the most used OS worldwide.

### Perspectives

Prior research has shown smartphone-based monitoring in young patients with psychiatric disorders to be feasible and acceptable (40), and as shown by us in a recent study, smartphone-based self-monitoring gives a valid reflection of clinical symptoms in young patients with newly diagnosed BD (41). However, self-monitoring is susceptible to attrition regardless of whether being paper based or electronic, and unless it is monitored many times a day, it cannot identify shorter fluctuations in symptoms during the day, which is found to be more common in young patients with BD (7, 42). Thus, automatically generated data could be a valuable supplement to smartphone-based self-assessment, which together gives a basis for a valid fine-grained tool for diagnosing and illness monitoring in young patients with newly diagnosed BD, delivering both subjective and more objective information.

## Conclusions

The present innovative pilot study investigated the use of automatically generated smartphone data collected daily during a long-term period in young patients with newly diagnosed BD and HC. Automatically generated smartphone data on social activity reflected clinically evaluated depressive and manic symptoms, differed between young patients with newly diagnosed BD and HC, and reflected self-reported changes in mood and activity in young patients with newly diagnosed BD. Although a rather low number of participants with a rather low level of affective symptoms during the study were included, our results suggest that automatically generated data on physical and social activity and phone usage could represent a potentially useful tool in diagnosing and monitoring young patients with newly diagnosed BD.

Future studies including young patients with newly diagnosed BD during more severe affective states, unaffected first-degree relatives, and different psychiatric disorders could provide interesting knowledge on early changes in communicative activities.

## Data Availability Statement

The datasets presented in this article are not readily available because the study is ongoing; and therefore, the research data are not shared. Requests to access the datasets should be directed to https://www.psykiatri-regionh.dk/forskning/forskningsomraader/Neuropsykiatri/cadic/Sider/default.aspx.

## Ethics Statement

The studies involving human participants were reviewed and approved by The Bipolar Illness Onset (BIO) study has been approved by the Ethics Committee in the Capital Region, Copenhagen, Denmark (Ref. Nr. H-7-2014-007) and the Danish Data Protection Agency, Capital Region of Copenhagen (Protocol No.: RHP-2015-023). Written informed consent to participate in this study was provided by the participants' legal guardian/next of kin. Written informed consent was obtained from the individual(s), and minor(s)' legal guardian/next of kin, for the publication of any potentially identifiable images or data included in this article.

## Author Contributions

LK, MF-J, and MV conceived the study and were in charge of overall direction and planning. JB and MF were in charge of the development of the technology, which was further adjusted for clinical use in by LK, MF-J, MV, MF, and JB. SM and SS were in charge of recruiting and clinical data collections. SM, SS, and MF-J conducted the statistical analysis. SM, MF-J, and LK were in charge of the manuscript. All authors have contributed in the final configuration of the manuscript.

## Conflict of Interest

LK has, within the preceding 3 years, been a consultant for Lundbeck. MV has in the last 3 years been a consultant for Sunovion, Janssen, and Lundbeck. JB and MF are co-founders and shareholders in Monsenso ApS. The remaining authors declare that the research was conducted in the absence of any commercial or financial relationships that could be construed as a potential conflict of interest.

## Publisher's Note

All claims expressed in this article are solely those of the authors and do not necessarily represent those of their affiliated organizations, or those of the publisher, the editors and the reviewers. Any product that may be evaluated in this article, or claim that may be made by its manufacturer, is not guaranteed or endorsed by the publisher.

## References

[1] Kessler RC Berglund P Demler O Jin R Merikangas KR Walters EE. Lifetime prevalence and age-of-onset distributions of DSM-IV disorders in the national comorbidity survey replication. Arch Gen Psychiatry. (2005) 62:593–602. 10.1001/archpsyc.62.6.593 15939837

[2] MacQueen GM Marriott M Begin H Robb J Joffe RT Young LT. Subsyndromal symptoms assessed in longitudinal, prospective follow-up of a cohort of patients with bipolar disorder. Bipolar Disord. (2003) 5:349–55. 10.1034/j.1399-5618.2003.00048.x 14525555

[3] Althubaiti A. Information bias in health research: definition, pitfalls, and adjustment methods. J Multidiscip Healthc. (2016) 9:211–7. 10.2147/JMDH.S104807 27217764 PMC4862344

[4] Kafali HY Bildik T Bora E Yuncu Z Erermis HS. Distinguishing prodromal stage of bipolar disorder and early onset schizophrenia spectrum disorders during adolescence. Psychiatry Res. (2019) 275:315–25. 10.1016/j.psychres.2019.03.051 30953877

[5] Vieta E Salagre E Grande I Carvalho AF Fernandes BS Berk M. Early intervention in bipolar disorder. Am J Psychiatry. (2018) 175:411–26. 10.1176/appi.ajp.2017.17090972 29361850

[6] Birmaher B Axelson D Strober M Gill MK Valeri S Chiappetta L. Clinical course of children and adolescents with bipolar spectrum disorders. Arch Gen Psychiatry. (2006) 63:175–83. 10.1001/archpsyc.63.2.175 16461861 PMC3079382

[7] Carlson GA Meyer SE. Phenomenology and diagnosis of bipolar disorder in children, adolescents, and adults: complexities and developmental issues. Dev Psychopathol. (2006) 18:939–69. 10.1017/S0954579406060470 17064424

[8] Statista. Number of Smartphone Users Worldwide from 2016 to 2021. (2020). Available online at: https://www.statista.com/statistics/330695/number-of-smartphone-users-worldwide/#statisticContainer (accesed April 2020).

[9] Beiwinkel T Kindermann S Maier A Kerl C Moock J Barbian G. Using smartphones to monitor bipolar disorder symptoms: a pilot study. JMIR Mental Health. (2016) 3:e2. 10.2196/mental.4560 26740354 PMC4720836

[10] Faurholt-Jepsen M Busk J Thornorarinsdottir H Frost M Bardram JE Vinberg M. Objective smartphone data as a potential diagnostic marker of bipolar disorder. Aust N Z J Psychiatry. (2019) 53:119–28. 10.1177/0004867418808900 30387368

[11] Faurholt-Jepsen M Vinberg M Frost M Christensen EM Bardram JE Kessing LV. Smartphone data as an electronic biomarker of illness activity in bipolar disorder. Bipolar Disord. (2015) 17:715–28. 10.1111/bdi.12332 26395972

[12] Faurholt-Jepsen M Vinberg M Frost M Debel S Margrethe Christensen E Bardram JE. Behavioral activities collected through smartphones and the association with illness activity in bipolar disorder. Int J Methods Psychiatr Res. (2016) 25:309–23. 10.1002/mpr.1502 27038019 PMC6860202

[13] Faurholt-Jepsen M Busk J Frost M Vinberg M Christensen EM Winther O. Voice analysis as an objective state marker in bipolar disorder. Transl Psychiatry. (2016) 6:e856. 10.1038/tp.2016.123 27434490 PMC5545710

[14] PewResearchCenter. Teens, Social Media & Technology 2018. PewResearchCenter (2018).

[15] Grunerbl A Muaremi A Osmani V Bahle G Ohler S Troster G. Smartphone-based recognition of states and state changes in bipolar disorder patients. IEEE J Biomed Health Inform. (2015) 19:140–8. 10.1109/JBHI.2014.2343154 25073181

[16] Linardon J Fuller-Tyszkiewicz M. Attrition and adherence in smartphone-delivered interventions for mental health problems: a systematic and meta-analytic review. J Consult Clin Psychol. (2020) 88:1–13. 10.1037/ccp0000459 31697093

[17] Faurholt-Jepsen M Frost M Christensen EM Bardram JE Vinberg M Kessing LV. The effect of smartphone-based monitoring on illness activity in bipolar disorder: the MONARCA II randomized controlled single-blinded trial. Psychol Med. (2019) 1–11. 10.1017/S0033291719000710 30944054

[18] Abdullah S Matthews M Frank E Doherty G Gay G Choudhury T. Automatic detection of social rhythms in bipolar disorder. J Am Med Inform Assoc. (2016) 23:538–43. 10.1093/jamia/ocv200 26977102 PMC11740758

[19] Palmius N Tsanas A Saunders KEA Bilderbeck AC Geddes JR Goodwin GM. Detecting bipolar depression from geographic location data. IEEE Trans Biomed Eng. (2017) 64:1761–71. 10.1109/TBME.2016.2611862 28113247 PMC5947818

[20] Stanislaus S Vinberg M Melbye S Frost M Busk J Bardram JE. Smartphone-based activity measurements in patients with newly diagnosed bipolar disorder, unaffected relatives and control individuals. Int J Bipolar Disord. (2020) 8:32. 10.1186/s40345-020-00195-0 33135120 PMC7604277

[21] Melbye S Kessing LV Bardram JE Faurholt-Jepsen M. Smartphone-based self-monitoring, treatment, and automatically generated data in children, adolescents, and young adults with psychiatric disorders: systematic review. JMIR Mental Health. (2020) 7:e17453. 10.2196/17453 33118950 PMC7661256

[22] Løventoft P Nørregaard L Frøkjær E. Designing Daybuilder: An Experimental App to Support People with Depression. (2012). p. 1–4.

[23] Niendam TA Tully LM Iosif A-M Kumar D Nye KE Denton JC. Enhancing early psychosis treatment using smartphone technology: a longitudinal feasibility and validity study. J Psychiatr Res. (2018) 96:239–46. 10.1016/j.jpsychires.2017.10.017 29126059

[24] Kessing LV Munkholm K Faurholt-Jepsen M Miskowiak KW Nielsen LB Frikke-Schmidt R. The bipolar illness onset study: research protocol for the BIO cohort study. BMJ Open. (2017) 7:e015462. 10.1136/bmjopen-2016-015462 28645967 PMC5734582

[25] Coello K Kjaerstad HL Stanislaus S Melbye S Faurholt-Jepsen M Miskowiak KW. Thirty-year cardiovascular risk score in patients with newly diagnosed bipolar disorder and their unaffected first-degree relatives. Aust N Z J Psychiatry. (2019) 53:651–62. 10.1177/0004867418815987 30518229

[26] Kjaerstad HL Mistarz N Coello K Stanislaus S Melbye SA Harmer CJ. Aberrant cognition in newly diagnosed patients with bipolar disorder and their unaffected relatives. Psychol Med. (2020) 50:1808–19. 10.1017/S0033291719001867 31456531

[27] Wing JK Babor T Brugha T Burke J Cooper JE Giel R. SCAN. Schedules for clinical assessment in neuropsychiatry. Arch Gen Psychiatry. (1990) 47:589–93. 10.1001/archpsyc.1990.01810180089012 2190539

[28] Bech P Gram LF Dein E Jacobsen O Vitger J Bolwig TG. Quantitative rating of depressive states. Acta Psychiatr Scand. (1975) 51:161–70. 10.1111/j.1600-0447.1975.tb00002.x 1136841

[29] Hamilton M. A rating scale for depression. J Neurol Neurosurg Psychiatry. (1960) 23:56–62. 10.1136/jnnp.23.1.56 14399272 PMC495331

[30] Young RC Biggs JT Ziegler VE Meyer DA. A rating scale for mania: reliability, validity and sensitivity. Br J Psychiatry. (1978) 133:429–35. 10.1192/bjp.133.5.429 728692

[31] Rosa AR Sanchez-Moreno J Martinez-Aran A Salamero M Torrent C Reinares M. Validity and reliability of the functioning assessment short test (FAST) in bipolar disorder. Clin Pract Epidemiol Ment Health. (2007) 3:5. 10.1186/1745-0179-3-5 17555558 PMC1904447

[32] Craig CL Marshall AL Sjostrom M Bauman AE Booth ML Ainsworth BE. International physical activity questionnaire: 12-country reliability and validity. Med Sci Sports Exerc. (2003) 35:1381–95. 10.1249/01.MSS.0000078924.61453.FB 12900694

[33] TheIPAQgroup. Guidelines for the Data Processing and Analysis of the International Physical Activity Questionnaire. (2005). Available online at: http://www.ipaq.ki.se/scoring.pdf (accesed April 2020).

[34] Bardram J Frost M Szántó K Faurholt-Jepsen M Vinberg M Kessing LV. Designing mobile health technology for bipolar disorder: a field trial of the monarca system. Paris: ACM. (2013) 2627–36. 10.1145/2470654.2481364

[35] Frost M Marcu G Hansen R Szaántó K Bardram JE editors. The MONARCA self-assessment system: Persuasive personal monitoring for bipolar patients. In: 2011 5th International Conference on Pervasive Computing Technologies for Healthcare (PervasiveHealth) and Workshops 2011 23-26 May. Dublin (2011).

[36] Zilka Gila C. Always with them: smartphone use by children, adolescents, and young adults-characteristics, habits of use, sharing, and satisfaction of needs. Univ Access Inform Soc. (2020) 19:145–55. 10.1007/s10209-018-0635-3

[37] Sahoo MK Chakrabarti S Kulhara P. Detection of prodromal symptoms of relapse in mania and unipolar depression by relatives and patients. Indian J Med Res. (2012) 135:177–83. 22446859 PMC3336848

[38] Burgdorf KS Simonsen J Sundby A Rostgaard K Pedersen OB Sørensen E. Socio-demographic characteristics of Danish blood donors. PLoS ONE. (2017) 12:e0169112. 10.1371/journal.pone.0169112 28182624 PMC5300150

[39] Golding J Northstone K Miller LL Davey Smith G Pembrey M. Differences between blood donors and a population sample: implications for case-control studies. Int J Epidemiol. (2013) 42:1145–56. 10.1093/ije/dyt095 23825379 PMC3781001

[40] Melbye S Kessing LV Bardram JE Faurholt-Jepsen M. Smartphone-based self-monitoring, treatment, and automatically generated data in children, adolescents, and young adults with psychiatric disorders: a systematic review. JMIR Ment Health. (2020) 7:e17453. 10.2196/preprints.17453 33118950 PMC7661256

[41] Melbye SA Stanislaus S Vinberg M Frost M Bardram JE Sletved K. Mood, activity, and sleep measured *via* daily smartphone-based self-monitoring in young patients with newly diagnosed bipolar disorder, their unaffected relatives and healthy control individuals. Eur Child Adolesc Psychiatry. (2021) 30:1209–21. 10.1007/s00787-020-01611-7 32743692 PMC8310852

[42] Stone AA Shiffman S Schwartz JE Broderick JE Hufford MR. Patient compliance with paper and electronic diaries. Control Clin Trials. (2003) 24:182–99. 10.1016/S0197-2456(02)00320-3 12689739

